# Protection of the heart by treatment with a divalent-copper-selective chelator reveals a novel mechanism underlying cardiomyopathy in diabetic rats

**DOI:** 10.1186/1475-2840-12-123

**Published:** 2013-08-28

**Authors:** Lin Zhang, Marie-Louise Ward, Anthony RJ Phillips, Shaoping Zhang, John Kennedy, Bernard Barry, Mark B Cannell, Garth JS Cooper

**Affiliations:** 1 School of Biological Sciences, Faculty of Science, University of Auckland, Private Bag 92019, Auckland 1142, New Zealand; 2 Department of Physiology, Faculty of Medical and Health Sciences, University of Auckland, Auckland, New Zealand; 3 Department of Surgery, Faculty of Medical and Health Sciences, University of Auckland, Auckland, New Zealand; 4 National Isotope Centre, GNS Science, Gracefield, Wellington, New Zealand; 5 Centre for Advanced Discovery and Experimental Therapeutics, Manchester Biomedical Research Centre, University of Manchester, Manchester, UK; 6 Department of Pharmacology, Medical Sciences Division, University of Oxford, Oxford, UK; 7 School of Physiology & Pharmacology, Medical Sciences Building, University of Bristol, Bristol, BS8 1TD, UK

**Keywords:** Copper homeostasis, Calcium homeostasis, Anti-oxidant defence, Cardiac contraction, Cardiovascular disease, Copper deficiency, Copper excess, Cardiomyopathy, Diabetes mellitus, Essential trace nutrient, Experimental therapeutics, Left-ventricular dysfunction, Left-ventricular remodelling, Calcium responsiveness, Myocardium, Myocardial calcium sensitivity, QT interval, Triethylenetetramine, Troponin

## Abstract

**Background:**

Intracellular calcium (Ca^2+^) coordinates the cardiac contraction cycle and is dysregulated in diabetic cardiomyopathy. Treatment with triethylenetetramine (TETA), a divalent-copper-selective chelator, improves cardiac structure and function in patients and rats with diabetic cardiomyopathy, but the molecular basis of this action is uncertain. Here, we used TETA to probe potential linkages between left-ventricular (LV) copper and Ca^2+^ homeostasis, and cardiac function and structure in diabetic cardiomyopathy.

**Methods:**

We treated streptozotocin-diabetic rats with a TETA-dosage known to ameliorate LV hypertrophy in patients with diabetic cardiomyopathy. Drug treatment was begun either one (*preventative protocol*) or eight (*restorative protocol*) weeks after diabetes induction and continued thereafter for seven or eight weeks, respectively. Total copper content of the LV wall was determined, and simultaneous measurements of intracellular calcium concentrations and isometric contraction were made in LV trabeculae isolated from control, diabetic and TETA-treated diabetic rats.

**Results:**

Total myocardial copper levels became deficient in untreated diabetes but were normalized by TETA-treatment. Cardiac contractility was markedly depressed by diabetes but TETA prevented this effect. Neither diabetes nor TETA exerted significant effects on peak or resting [Ca^2+^]_i_. However, diabetic rats showed extensive cardiac remodelling and decreased myofibrillar calcium sensitivity, consistent with observed increases in phosphorylation of troponin I, whereas these changes were all prevented by TETA.

**Conclusions:**

Diabetes causes cardiomyopathy through a copper-mediated mechanism that incorporates myocardial copper deficiency, whereas TETA treatment prevents this response and maintains the integrity of cardiac structure and myofibrillar calcium sensitivity. Altered calcium homeostasis may not be the primary defect in diabetic cardiomyopathy. Rather, a newly-described copper-mediated mechanism may cause this disease.

## Background

Cardiovascular disease (CVD) is the main cause of mortality in diabetic patients, in whom it is frequently accompanied by heart failure [1]. About three-quarters of the CVD risk in diabetes may be attributable to hypertension [2]. Other significant contributors to risk include obesity, atherosclerosis, dyslipidaemia, endothelial dysfunction, defective platelet function, coagulation abnormalities, and diabetic cardiomyopathy [2]. The latter condition is considered to be a diabetes-specific cardiomyopathy, which is independent of macrovascular and microvascular disease and which contributes substantively to morbidity and mortality [2-4].

Effective treatments for established heart failure in diabetes are limited [4,5] and its prognosis is particularly poor in patients with type-2 diabetes (T2D) [6]. Elevated risk persists despite best available treatments with existing classes of medications, so new and improved therapeutic approaches for heart failure in diabetes are required.

Copper is an essential trace element that plays key roles in many vital copper enzymes, e.g. cytochrome c oxidase, caeruloplasmin, copper/zinc superoxide dismutase (SOD1), and lysyl oxidase [7]. Free divalent copper, Cu(II), is the most redox-active metal ion in mammalian tissues and excess can cause tissue damage by generating highly-reactive hydroxyl radicals [8], which inactivate essential enzymes and cause lipid peroxidation and DNA damage, thereby disrupting cell metabolism [9,10].

Insufficient dietary copper intake can cause systemic copper deficiency, myocardial copper deficiency, hypertrophic cardiomyopathy, and heart failure [11-14], all of which are reversible by restoration of normal copper intake [15]. Moreover, there is evidence that dietary copper supplementation reversed hypertrophic cardiomyopathy induced by chronic pressure overload in mice consuming a normal-copper diet; in that study, LV copper levels fell by ~20% as a result of the pressure overload [16], but systemic copper balance was not reported.

Copper accumulation in excess of bodily requirements is also cytotoxic [17,18]. Altered tissue copper levels have sometimes been reported to accompany diabetes in patients or animal models [19,20]. We have shown, however, in a phase-2 trial that employed a balance study, that T2D patients acquire substantive copper overload, and that they do not demonstrate systemic signs of copper deficiency. Copper regulation in diabetic patients is thus clearly differentiated from the copper deficiency states that occur in animals and, rarely, in patients [21,22]. The significance of dysregulated copper in diabetes-evoked heart disease was substantively strengthened when we showed that the Cu(II)-selective chelator, TETA can restore cardiac structure and function in diabetic patients and animals, without concomitant lowering of blood glucose or systemic blood pressure [23,24].

T2D patients acquire a hyperglycaemia-driven pathogenic abnormality of copper homeostasis that is reversible by TETA-treatment [25], which also restores LV mass in patients with LV hypertrophy (LVH) [24]. TETA is a highly-selective divalent copper chelator [25,26], which is used as a second-line treatment for Wilson’s disease [27]. In rats used as a model of diabetic cardiomyopathy, TETA causes cardiac regeneration [23], reversal of LV dysfunction [28], and improved antioxidant defences [23,29]. In aggregate, these findings provide substantive support for the idea that defective copper regulation plays a key role in the pathogenesis of cardiomyopathy and LV dysfunction in diabetes [22]. However, the molecular basis for this linkage remains uncertain, and motivated the current study. The evidence linking defective copper regulation to the pathogenesis of the diabetic complications in the heart, kidneys, arteries and nerves has recently been reviewed [21,22].

Defects in intracellular Ca^2+^ homeostasis have been implicated in the impaired mechanical performance of the diabetic heart [4,30-32] but there is no current consensus from published studies, where resting Ca^2+^ has been reportedly decreased [33-35], increased [36,37], or unchanged [38,39]. Similarly, the amplitude of the Ca^2+^ transient has also been reported as decreased [35,40-42], increased [36], or unchanged [43,44]. Therefore, we re-examined the changes in Ca^2+^ homeostasis underlying diabetic cardiomyopathy in the current study. We also determined whether TETA exerts its inotropic effects by modifying intracellular Ca^2+^ homeostasis, since copper dysregulation can generate reactive oxidative species and cause defects in anti-oxidant defences which could further accentuate abnormalities in intracellular Ca^2+^ regulation [45,46].

Here we have tested the hypothesis in rats used as a model of the diabetic complications that diabetes might cause linked abnormalities of copper and calcium homeostasis, which could interact to yield cardiomyopathy and LV dysfunction.

## Methods

*Animals* This study was approved by the University of Auckland Animal Ethics Committee and performed according to the Guide for the Care and Use of Laboratory Animals [47] and the ARRIVE guidelines for the reporting of animal research [48].

*Reagents* All chemical reagents were obtained from Sigma and were of analytical grade or better, unless otherwise stated.

*Induction and verification of diabetes in rats* Diabetes was induced in adult male Wistar rats (200–250 g body-wt; 6–7 weeks of age) by a single intravenous injection of STZ (60 mg/kg body-wt dissolved in 0.9% saline) as described [32]; control rats received 0.9% saline only. Diabetes was diagnosed by demonstration of sequential tail-vein blood glucose concentrations >11 mmol/L [23]. TETA disuccinate (CarboGen, Switzerland; 30 mg/day-rat) in the drinking water (18 MΩ; MilliQ, Millipore, Billerica, MA) [49] was begun either one week after injection and continued for a further 7 weeks (*preventative protocol*, [32]), or 8 weeks after injection and continued for a further 8 weeks (*restorative protocol*[23]). Throughout the study, animals were housed (in pairs) in a temperature-controlled unit with a fixed 12-hr light/dark cycle, and received *ad libitum* access to food and water.

*Rationale for choice of drug dosage* We based the choice of TETA dosage used here on those employed in known clinical applications of TETA (TETA dihydrochloride or trientine) in the treatment of Wilson’s disease, and for the experimental therapy of diabetes. Dosages employed for the treatment of Wilson’s disease in adults typically vary from 750–2000 mg/day (equivalent to ~10.7-28.6 mg/kg-day in 70-kg adults) [50]. Here, we administered TETA disuccinate (a new salt form with improved stability characteristics [49]) in the drinking water to diabetic rats at 30 mg/day (equivalent to ~17 mg/day-rat or ~68 mg/kg-day of trientine in 250-g rats). This dosage is supported by our published dose-rising phase-1 clinical trial, where we showed that dosages of 1200 and 3600 mg/day (equivalent to ~17 and 51 mg/kg-day in 70-kg adults) were effective and well tolerated in healthy human volunteers [51] and also by our published phase-2 trial where 1200 mg/day of trientine (~17 mg/kg-day in 70-kg adults) administered for 12 months markedly improved LV mass in T2D patients with LVH [24].

*Measurement of myocardial copper levels* was performed by applying particle-induced X-ray emission spectroscopy (PIXE) with Rutherford backscattering spectrometry (RBS) in freeze-dried sections of *ex vivo* LV myocardium [52] according to established methods [53]. The calibration, measurements and limits of detection were based on the areas of the *Kα* x-ray peaks as assessed by the software package GUPIX Elemental, and concentrations were extracted from PIXE spectra using GUPIX software (gupix@listserv.uoguelph.ca).

*Force and [Ca*^*2+*^*]*_*i*_*in left ventricular trabeculae* were measured as previously described [54]. Briefly, unbranched and cylindrical trabeculae were dissected free from the underlying LV tissue and mounted on a force transducer (AE801, SensorOne, Sausalito CA) on the stage of an inverted microscopy (Nikon Diaphot 300, Japan). The trabeculae were perfused with Krebs-Henseleit (KH) solution containing (in mmol/L): NaCl, 118; KCl, 4.75; KH_2_PO_4_, 1.18; MgSO_4,_ 1.18; NaHCO_3,_ 24.8; and D-glucose, 10; with BDM, 20; CaCl_2_, 0.5; gassed with O_2_:CO_2_ 95:5 [vol/vol]. Once the trabeculae were producing stable stimulus-evoked twitches, they were loaded with the fluorescent Ca^2+^ indicator fura-2/AM (10 μmol/L, 2-hr; Invitrogen, Carlsbad, CA). Force and [Ca^2+^]_i_ were then measured simultaneously at a fixed sarcomere length (2.1-2.2 μm), either at 37°C, 5-Hz, and 1.5 mmol/L [Ca^2+^]_o_, or other experimental conditions. The ratio of the emitted fluorescence at the two excitation wavelengths (340/380 ratio) of fura-2 was obtained (Cairn Research, Faversham, Kent, UK) as a measurement of [Ca^2+^]_i_. The force measurements were normalized by the trabecular cross-sectional area and expressed as stress (mN/mm^2^).

The steady-state force-[Ca^2+^]_i_ relationship was evaluated in LV trabeculae treated with 5 μmol/L of ryanodine, a plant alkaloid considered to block ryanodine receptor channels to generate a long-lived subconductance (half-open) state [55] and eventually to deplete the sarcoplasmic-reticular calcium stores. The drug effect reached steady state after 30-min exposure, whereupon fused tetani could be reliably elicited by rapid stimulation (12 Hz) for 6 s at a series of [Ca^2+^]_o_ values from 0.5 mmol/L to the concentration where the maximum force was obtained. Tetanus at different [Ca^2+^]_o_ was separated by a “resting” period of 2 min. To avoid precipitation at high Ca^2+^ concentrations, solutions used in these experiments were switched to a phosphate-free composition including (in mmol/L): NaCl, 108; KCl, 5; MgCl_2,_ 1; HEPES, 5; NaCH_2_COOH, 20; and D-glucose, 10. Calibration in terms of absolute values of [Ca^2+^]_i_ and data analysis were performed following published procedures [32,54].

*Measurement of caffeine-induced Ca*^*2+*^* transients in isolated cardiomyocytes* LV cardiomyocytes were isolated by enzymatic digestion with 1 mg/ml collagenase Type-II (Worthington, NJ, USA) and 0.1 mg/ml proteinase type-XXIV (Sigma, MO, USA) as previously described [56]. The yield of the isolation was usually around ~80%. Cardiomyoctes were loaded with fura-2/AM (5 μmol/L, 10 min; Invitrogen). Rod-shaped quiescent cardiomyocytes with sharp edges and clear striations were used in the investigation. Caffeine-induced Ca^2+^ transients were recorded from cardiomyocytes isolated from each group of rats, which were exposed to a series of solution changes. Firstly, cells were stimulated at 1 Hz in normal Tyrode’s solution (in mmol/L): HEPES, 10; NaCl, 143; KCl, 5.4; MgCl_2_, 0.5; glucose, 10; taurine, 20; and CaCl_2_, 1.5; pH 7.4 finally adjusted with NaOH. Then, following 30-s perfusion with Tyrode’s solution with 0 mmol/L [Ca^2+^]_o_ and without stimulation, 10 mmol/L caffeine was added (to functionally eliminate Ca^2+^ storage in the SR), and Ca^2+^ transients were recorded. After the [Ca^2+^]_i_ had decreased to the resting level, cells were perfused with Tyrode’s solution incorporating 1.5 mmol/L [Ca^2+^]_o_ and stimulated at 1 Hz (to restore the SR Ca^2+^) followed by a further series of solution changes in the following order: 0 mmol/L [Ca^2+^]_o,_ 30 s; and then 10 mmol/L caffeine with 0 mmol/L [Na^+^]_o_ and 10 mmol/L [Ni^2+^]_o_ (to eliminate both the SR Ca^2+^ store and sarcolemmal Ca^2+^ extrusion via the Na/Ca exchanger (NCX). Ca^2+^ transients were continuously recorded.

*Western Blotting* was performed with proteins extracted from LV tissue as described [32]. Briefly, frozen LV tissue was homogenized in ice-cold lysis buffer (50 mmol/l Tris–HCl, pH 8; 150 mmol/l NaCl; 1% NP-40; 0.5% sodium deoxycholate; and 0.1% sodium dodecylsulphate) with a proteinase inhibitor mixture and a phosphatase inhibitor mixture (Roche, Indianapolis, IN), and then centrifuged at 13,000 *g* for 1 h at 4°C. Protein concentration in the supernatant was determined with a bicinchoninic acid protein assay (Pierce, Rockford, IL). Western blots were performed by using a rabbit anti-NCX antibody (Ab, 1:500, Swiss Antibodies, Swant, Switzerland), a mouse anti-SERCA2a antibody (1:1000, Affinity BioReagents, Golden, CO, USA), a mouse anti-TnT antibody (1:1000, Abcam, Cambridge, UK), and a mouse anti-TnI antibody (1:1000, Abcam). Specific signals were detected using anti-rabbit or anti-mouse IgG-horseradish peroxidase conjugate and ECL-Plus Western Blotting Detection Reagents (GE Healthcare, Little Chalfont, Buckinghamshire, UK) according to the manufacturer's instructions. An LAS 3000 image reader (Fuji Photo Film Co. Ltd, Tokyo, Japan) was used to detect signals and take images of the membranes. Phosphoproteins were detected by using Pro-Q Diamond phosphoprotein gel-stain followed by SYPRO^®^ Ruby total protein gel-stain (Invitrogen) and signals were detected by a Pharos FX™ Plus Molecular Imager (BioRad, CA). Afterwards, all images were analysed using Multi Gauge analysis software (Fuji Photo Film).

*Immunohistochemistry of LV free wall* was performed as described [32]. Briefly, sectioned cardiac tissues (30 μm) were immunolabelled with rabbit anti-rat type-I or type-III collagen antibody (1:100 in 5% goat serum/1% BSA/PBS; Chemicon, Boronia, Australia), and then with secondary antibody (1:200) (Alexa Fluor 488 goat anti-rabbit Ab; Invitrogen) and phalloidin-rhodamine (1:100, Invitrogen). Slides were imaged with a confocal microscope (Zeiss LSM410). F-actin and collagen types-I and -III were quantified using a custom-written IDL analysis program based on signal-thresholding.

*Statistical analysis* Data are expressed as mean ± SE. Differences between means were analysed by using analysis of variance (ANOVA) with appropriate *post-hoc* tests. Statistical difference was considered significant at *P* < 0.05. Normality and equality of variance of samples were tested by applying the Shapiro-Wilk test or Spearman rank correlation, respectively. If either of these two tests failed, then Kruskal-Wallis one-way ANOVA with Dunn’s multiple comparisons test was used for the statistical analysis. Otherwise, one-way ANOVA with the *post-hoc* Holm-Sidak method or Tukey’s *post-hoc* test were used as appropriate.

## Results

*Metabolic responses to drug treatment* TETA had no effect on the elevated blood glucose levels in diabetic rats, although it significantly increased the body-weight of diabetic rats in the latter stages of preventative treatment (Week 8; *P* = 0.02, Table 1); these findings could imply the occurrence of a systemic/metabolic improvement in TETA-treated animals without improved glycaemic regulation.

**Table 1:** Descriptive variables on the experimental day (at week 8)

	**Control**	**Diabetic**	**TETA-treated diabetic**	***P***
**Body-weight (g)**	438 ± 7 (28)	247 ± 8 (28) ^a^	269 ± 5 (26) ^b, c^	<0.0001
**Heart-weight (g)**	1.60 ± 0.07 (24)	1.24 ± 0.06 (24) ^a^	1.25 ± 0.05 (20) ^c^	<0.0001
**Tibial length (cm)**	4.23 ± 0.04 (13)	3.86 ± 0.06 (16) ^a^	3.87 ± 0.06 (20) ^c^	<0.0001
**Heart weight/Body-weight (x 10**^**3**^**)**	3.7 ± 0.1 (23)	5.3 ± 0.2 (21) ^a^	5.0 ± 0.2 (20) ^c^	<0.0001
**Heart weight/ (Tibial length)**^**2 **^**(mg/cm**^**2**^**)**	94 ± 4 (13)	90 ± 5 (13)	84 ± 3 (20)	0.23
**Blood glucose (mmol/L)**	4.7 ± 0.1 (24)	30.8 ± 0.8 (30) ^a^	32.2 ± 0.7 (26) ^c^	<0.0001
**Heart rate (min**^**-1**^**)**	390 ± 9 (11)	311 ± 6 (18) ^a^	315 ± 12 (10) ^c^	<0.0001
**Corrected QT interval (ms)**	177 ± 4 (11)	192 ± 4 (18) ^a^	138 ± 14 (10) ^b^	0.001

Data are means ± SEM. Numbers of animals were as shown in parentheses. *P*-values are by Kruskal-Wallis one-way ANOVA with Dunn’s multiple-comparisons test or one-way ANOVA with the Holm-Sidak method as appropriate; ^a^, ^b^, ^c^, significant differences: ^a^, Control vs Diabetic groups; ^b^, Diabetic vs TETA-treated diabetic; ^c^, Control vs TETA-treated Diabetic.

*TETA-treatment restored total copper content in LV myocardium of diabetic rats* After 16 weeks (*restorative protocol* as per Methods [23]), the total copper content of the LV myocardium was much lower in diabetic animals than in controls (diabetic: 29 ± 2 μg/g dry LV tissue, *n =* 7; control: 50 ± 6 μg/g, *n =* 7, *P* = 0.030), whereas in TETA-treated diabetic animals, total copper was fully normalized (TETA-treated diabetic: 48 ± 6 μg/g, *n =* 7, *P* = 0.032 vs untreated diabetic).

*TETA prevented diabetes-evoked QTc prolongation* Untreated diabetes caused significant slowing of the heart rate compared with control values (*P* < 0.0001), and TETA did not modify this effect. However, QT intervals corrected for heart rate (QT_C_) were prolonged in diabetic rats whereas TETA prevented the development of QT_C_ prolongation (Table 1). By contrast, TETA treatment did not modify QTc values in non-diabetic rats (results not shown).

These data are consistent with the idea that TETA-mediated prevention of QTc prolongation resulted from normalization of myocardial copper regulation, pointing to the existence of copper-mediated mechanisms that contribute to QTc regulation.

**Figure 1:**
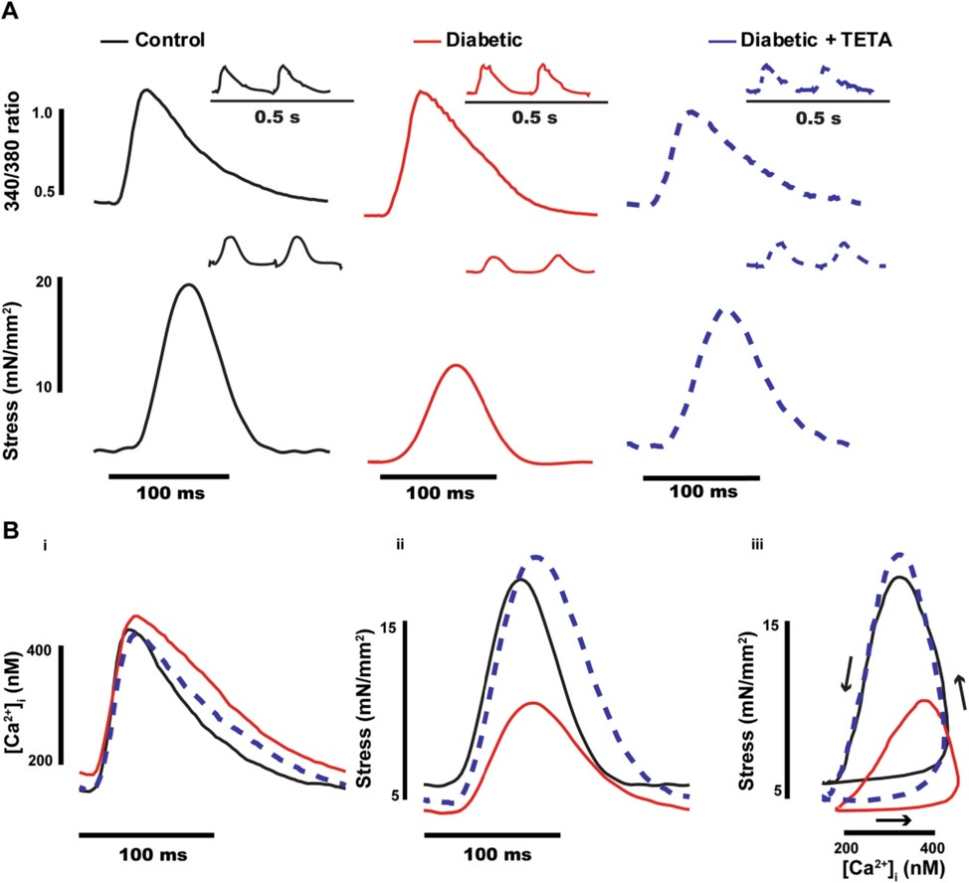
**Isometric force and [Ca**^**2+**^**]**_**i **_**measured from LV trabeculae.** Isometric force and [Ca^2+^]_i_ were measured simultaneously in LV trabeculae at 37°C, 5 Hz stimulation frequency and 1.5 mmol/L [Ca^2+^]_o_, conditions that are close to physiological. **(A)** Exemplary traces of Ca^2+^ transients (fura-2/AM 340/380 ratio) and corresponding isometric stress (force normalized to the corresponding muscle’s cross-sectional area) averaged over a number of cardiac cycles in representative trabeculae from the 3 experimental groups, which typify those used for data analysis. Inserted figures are corresponding raw traces of Ca^2+^ transients and corresponding isometric stress. **(B)** Averaged values from 7 trabeculae in each experimental group for (i) the Ca^2+^ transient, (ii) isometric stress and (iii) phase plots of Ca^2+^ transients and corresponding isometric stress. Diabetic rats showed decreased responsiveness to Ca^2+^, as indicated by the rightward shift of the relaxation phase (iii) and TETA-treatment prevented development of this defect.

*TETA prevented the diabetes-evoked deterioration of cardiac contractility* Diabetic rats showed depressed peak stress compared to controls (*P* = 0.02; Figures 1Bii, 2Aiii) but this response was prevented by TETA (*P* = 0.04). Resting stress was not significantly different between experimental groups (Figures 1Bii, 2Aiv). Neither diabetic status nor TETA exerted significant effects on the levels of peak/or resting [Ca^2+^]_i_ (Figures 1Bi, 2Ai, ii). The relaxation-phase of isometric stress plotted against [Ca^2+^]_i_ showed a rightward-shift in diabetic rats, consistent with decreased myofibrillar Ca^2+^ sensitivity [57], whereas this effect was fully prevented by TETA (Figure 1Biii).

**Figure 2:**
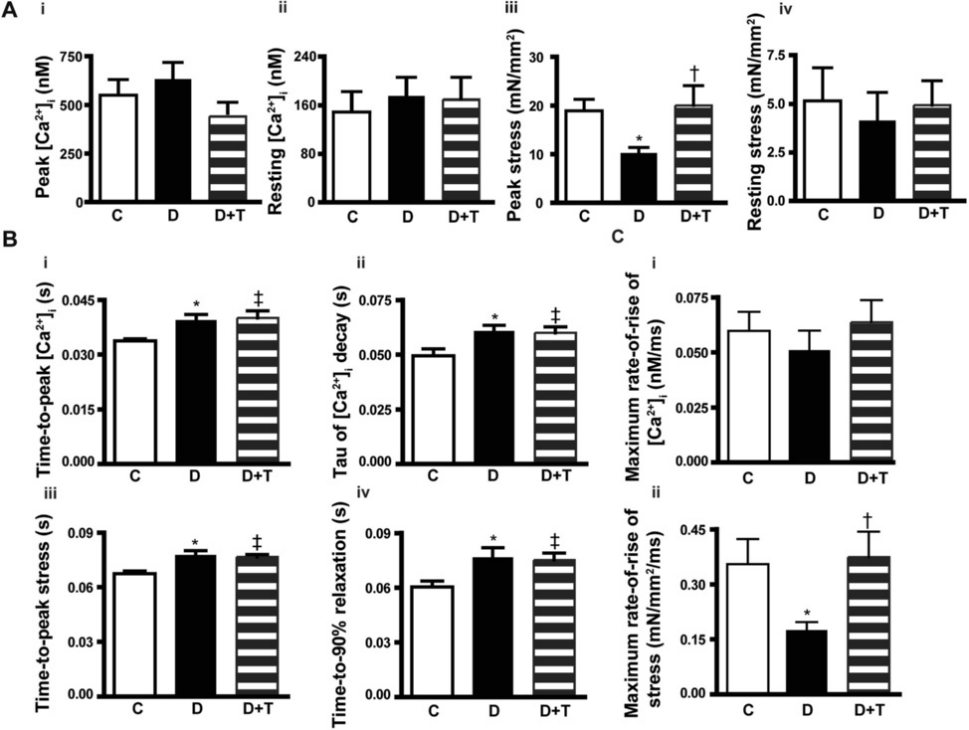
**Depressed cardiac contractility in diabetic rats prevented by TETA-treatment.** Depressed cardiac contractility in diabetic rats was prevented by TETA-treatment, which by contrast did not modify diabetes-induced alterations in [Ca^2+^]_i_ homeostasis. **(A)** Diabetic rats showed unchanged peak (i) and resting (ii) [Ca^2+^]_i_ values but had concomitantly decreased peak stress (iii) (Diabetic: 10 ± 1 mN/mm^2^; Control: 17 ± 2 mN/mm^2^, *P =* 0.02) and unchanged resting stress (iv). TETA-treatment preserved peak stress in diabetic rats (TETA-treated diabetic: 20 ± 4 mN/mm^2^, *P =* 0.04) but did not significantly modify peak or resting [Ca^2+^]_i_. **(B)** The time course of the Ca^2+^ transient was prolonged in diabetic rats: (time-to-peak [Ca^2+^]_i_ (i); and time constant of decay of the Ca^2+^ transient (ii)), as was the time course of isometric stress (time-to-peak stress (iii); and time-to-90% relaxation (iv)). However, TETA-treatment had no effect on the time course of either variable. **(C)** The maximum rate of rise in the Ca^2+^ transient was unchanged (i) whereas the maximum rate of stress development was decreased in diabetic rats (ii), and this decrease was prevented by TETA-treatment. C: Control (Open bars, *n =* 10); D: Diabetic (Solid bars, *n =* 8); D + T: TETA-treated diabetic (Patterned bars, *n =* 7). Data are means ± SEM, one-way ANOVA with application of the *post-hoc* Holm-Sidak test: * C vs D; ‡ C vs D + T; † D vs D + T; *P* < 0.05.

Although peak and resting levels of [Ca^2+^]_i_ were maintained in all experimental animals, diabetic rats had a prolonged time-course of their Ca^2+^ transients (increased time-to-peak [Ca^2+^]_i_ and time-constant of decay) compared to controls (both *P* = 0.02, Figure 2Bi, ii). Consistently, the time-course of contraction in diabetic rats was also slower (increased time-to-peak stress and time-to-90 % relaxation, *P* = 0.001 and *P* = 0.025, respectively, Figure 2Biii, iv). Moreover, TETA had no effect on the prolonged time-course of either the contraction or the Ca^2+^ transient in diabetic rats.

**Figure 3:**
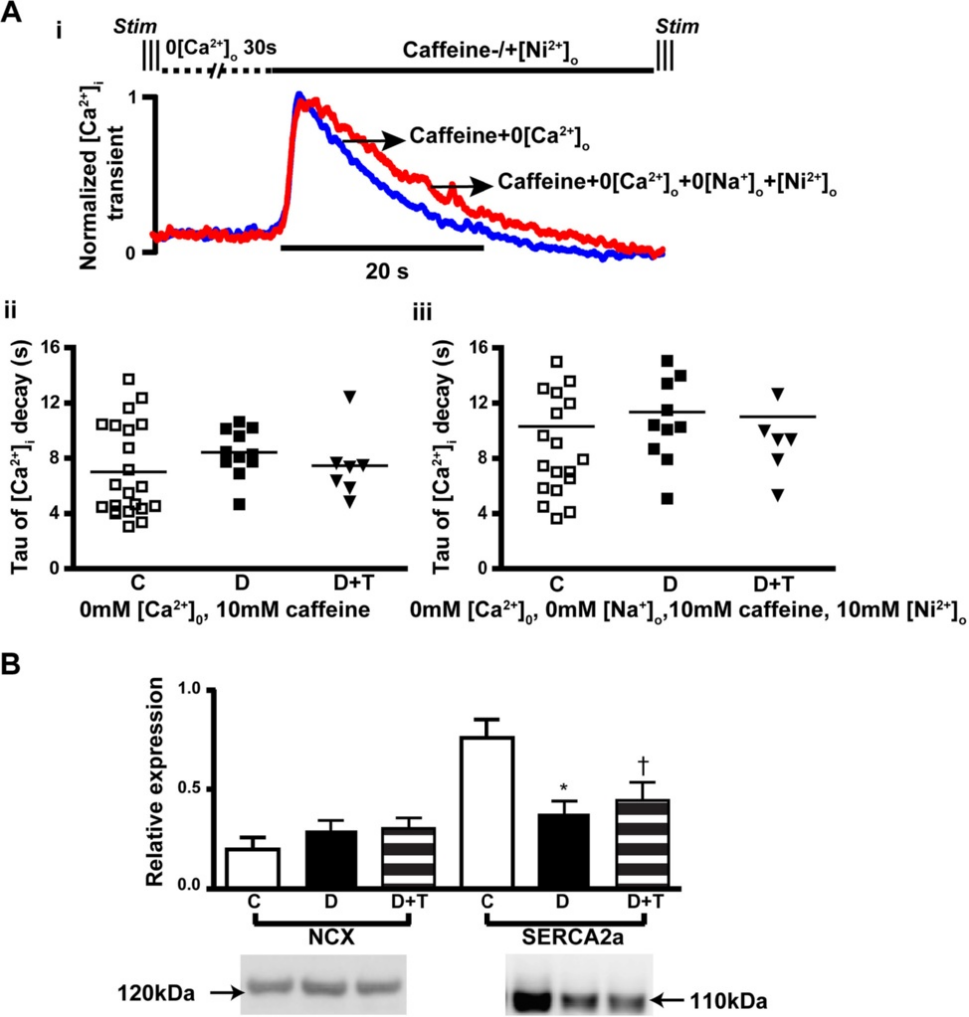
**Alterations in SERCA2a and NCX in LV myocardium. (A)** Caffeine-induced Ca^2+^ transients were recorded from cardiomyocytes isolated from each group of rats, which were exposed to a series of solution changes as described in methods. (i) shows examples of normalized caffeine-induced Ca^2+^ transients from a single cell, with pooled data shown in (ii)&(iii). There was no significant difference in the time-constant of decay of caffeine-induced Ca^2+^ transients among groups in either of the caffeine perfusion solutions, indicating no change of NCX activity among groups. These data suggest that the slower decay of the Ca^2+^ transient in diabetic rats did not arise from differences in NCX function. Consistently, a western blotting study **(B)** showed no significant change in NCX levels (molecular weight 120 kDa, n = 8 in each group) among groups, but decreased expression of SERCA2a (molecular weight 110 kDa, n = 7 in each group) in diabetic rats. TETA had no effect on levels of either transporter. C: Control (Open bars); D: Diabetic (Solid bars); D + T: TETA-treated diabetic (Patterned bars). Data are means ± SEM, one-way Kruskal-Wallis ANOVA with *post-hoc* application of Dunn’s Multiple Comparisons test (*P =* 0.0007): * C vs D, *P* < 0.05; † C vs D + T, *P* < 0.05.

Decay of the Ca^2+^ transient in the rat is dominated by the rate of Ca^2+^ uptake by the sarcoplasmic reticulum via SERCA2a and, to a lesser extent by sarcolemmal Ca^2+^ extrusion via the Na/Ca exchanger (NCX, ~7-9% of total) [58]. Caffeine-induced Ca^2+^ transients were recorded and analyzed from LV cardiomyocytes to assess the activity of SERCA2a and NCX in the experimental groups. 10 mmol/L caffeine was used to eliminate the SR storage of Ca^2+^ during the decay of the Ca^2+^ transient, while 10 mmol/L [Ni^2+^]_o_ and 0 mmol/L [Na^+^]_o_ were used to further eliminate the contribution of NCX to the decay of the Ca^2+^ transient. Results (Figure 3A) showed no significant difference in the time-constant of decay of caffeine-induced Ca^2+^ transients among groups in any of the perfusion solutions. In comparison with the slower time-constant of decay of normal Ca^2+^ transients in diabetic rats, the unaltered caffeine-induced Ca^2+^ transients indicate that SERCA2a activity was down-regulated whereas NCX activity was unchanged. These findings are consistent with the Western-blotting study (Figure 3B), in which diabetic rats showed lower levels of SERCA2a (*P* = 0.01), whereas NCX was unaltered. These results could explain the slower decay of the Ca^2+^ transient in diabetic trabeculae. TETA had no significant effect on the expression of these proteins, consistent with the unchanged decay-rate of the Ca^2+^ transient in TETA-treated diabetic rats.

The unchanged levels of peak and resting [Ca^2+^]_i_ coupled with the slower kinetics of the Ca^2+^ transient cannot explain the depressed cardiac contractility in diabetic rats [32], since these alterations would favour increased force production, which was not observed here. We therefore hypothesized that altered [Ca^2+^]_i_ handling may not be the primary contributor to diabetes-evoked cardiac dysfunction, and that diabetic rats may have intrinsic myocardial defects that contribute to their depressed cardiac contractility. Our observations invited further investigation of myofibrillar Ca^2+^ sensitivity and cardiac remodelling in diabetic rat hearts.

**Figure 4:**
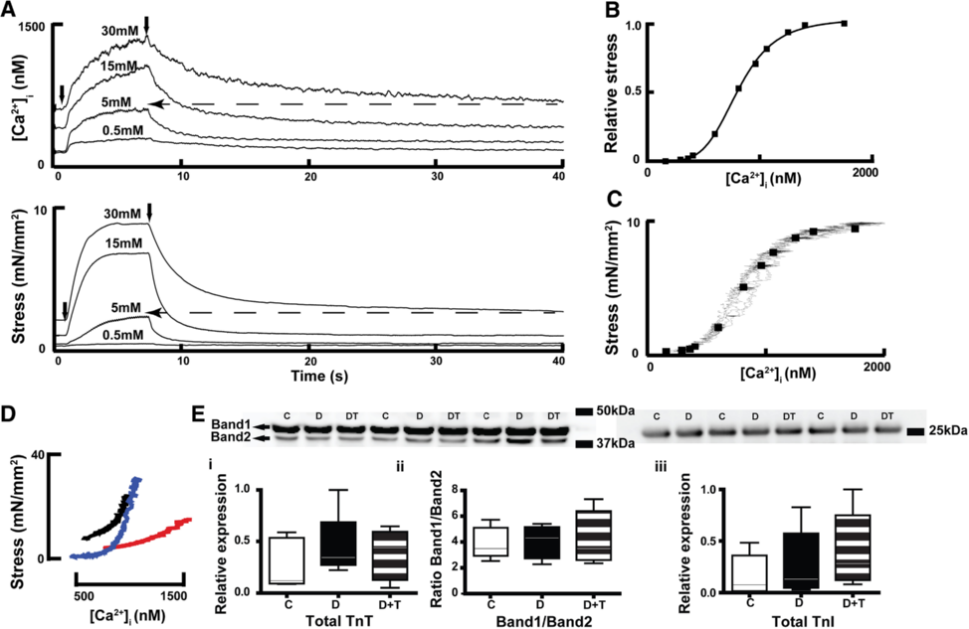
**The steady-state force-[Ca**^**2+**^**]**_**i **_**relationship and expression of TnT** &**TnI in LV myocardium. (A)** Representative traces of [Ca^2+^]_i_ and stress during tetanic stimulation of a trabecula from a diabetic rat (at [Ca^2+^]_o_: 0.5, 5, 15 and 30 mmol/L); the solid arrows indicate where stimulation started and ended; the dashed arrows indicate that the resting [Ca^2+^]_i_ and the corresponding resting stress at 30 mmol/L [Ca^2+^]_o_ were comparable to the tetanized [Ca^2+^]_i_ and its corresponding stress at 5 mmol/L [Ca^2+^]_o_. **(B)** The corresponding data obtained 4 s after commencing tetanic stimulation from this trabecula were fitted to the Hill equation as shown. **(C)** The rising aspects of the phase plots of [Ca^2+^]_i_ and tetanus at different [Ca^2+^]_o_ values from the same trabecula are shown (irregular grey lines), where the data used for fitting to the Hill equation (as in B) have been superimposed (black squares). **(D)** Averaged relaxation phase plots of [Ca^2+^]_i_ and tetanus at [Ca^2+^]_o_ 20 mmol/L from numbers of trabeculae in each experimental groups (Control: black line, *n =* 9; Diabetic: red line, *n =* 7; TETA-treated diabetic: blue line, *n =* 7). Diabetic rats showed a rightward shift of the relaxation phase, consistent with decreased myofibrillar Ca^2+^ sensitivity whereas TETA-treatment preserved the Ca^2+^ sensitivity in diabetic hearts. **(E)** Expression of TnT (upper left panel shows representative western blots of three animals from each group; box and whisker plots (median, range) below show normalized densitometry of both TnT bands (i) & ratios of the two TnT bands (ii) at molecular weights in the range of 40–42.5 kDa, n = 5 in each group); and TnI (right panel; iii, molecular weight 28 kDa, n = 6 in each group) in LV tissue from the three experimental groups; these showed no significant between-group differences. C: Control (Open bars); D: Diabetic (Solid bars); D + T: TETA-treated diabetic (Patterned bars). Significance was tested by one-way Kruskal-Wallis ANOVA.

*TETA prevented the development of abnormal myofibrillar Ca*^*2+*^*sensitivity in diabetic hearts* The rightward-shift in the relaxation component of the phase-plot of isometric stress and [Ca^2+^]_i_ in diabetic rats (Figure 1Biii), is consistent with decreased myofibrillar Ca^2+^ sensitivity. TETA prevented this rightward-shift, suggesting that myofibrillar Ca^2+^ sensitivity was preserved in the drug-treated diabetic animals, contributing to their normal cardiac contractility. However, the rapid change in the Ca^2+^ transient during a normal twitch does not favour full development of the interaction between myofilaments and [Ca^2+^]_i_. We therefore investigated the steady-state force-[Ca^2+^]_i_ relationship to assess the role of Ca^2+^ in modulating force production under these conditions. Trabeculae were treated with 5 μmol/L ryanodine and high stimulation-frequency (12 Hz, 6 s) to induce cardiac tetanus for investigation of the steady-state force-[Ca^2+^]_i_ relationship (Figure 4A) [59]. During each tetanus, stress plateaued 3–4 s after the onset of stimulation, while [Ca^2+^]_i_ increased throughout. Tetanized stress and [Ca^2+^]_i_ increased with increasing [Ca^2+^]_o_ until maximum stress was reached: that is, increase in stress ceased even if [Ca^2+^]_i_ continued to increase. Measurement of stress and [Ca^2+^]_i_ were obtained 4 s after stimulation onset for each [Ca^2+^]_o_ level and values were fitted with the Hill equation (*see* Figure 4B, pooled data summarized in Table 2). Diabetic rats showed depression of maximum Ca^2+^-activated contractility to ~47% of control values (*P* = 0.008) whereas TETA-treatment preserved the maximum contractility in treated-diabetic animals (*P* = 0.03). Untreated-diabetic rats also showed increased K_1/2_ (the value of [Ca^2+^]_i_ when half the maximum stress was reached), consistent with decreased myofibrillar Ca^2+^ sensitivity (*P* = 0.012). TETA prevented this alteration of K_1/2,_ suggesting that drug-treated diabetic rats have normal myofibrillar Ca^2+^ sensitivity compared to untreated ones (*P* = 0.05). Furthermore, the phase-plot between [Ca^2+^]_i_ and tetanus at 20 mmol/L [Ca^2+^]_o_ from a number of trabeculae in each experimental group (Figure 4D) demonstrated a right-shifted relaxation phase in diabetic rats consistent with decreased myofibrillar Ca^2+^ sensitivity, which was prevented by TETA.

**Table 2:** Values obtained by fitting the Hill equation to the stated number of trabeculae in each group

	**Control**	**Diabetic**	**TETA-treated diabetic**
**Number**	9	7	7
**Maximum stress (mN/mm**^**2**^**)**	38 ± 4	18 ± 4 ^a^	39 ± 8 ^b^
**Hill coefficient (N)**	6.3 ± 1.2	5.1 ± 0.9	8.4 ± 1.6
**K**_**1/2 **_**(nmol/L)**	688 ± 60	1018 ± 105 ^c^	799 ± 37

Data are means ± SEM. *P-*values were determined by Kruskal-Wallis one-way ANOVA with *post-hoc* Dunn’s Multiple-Comparisons tests: ^a^, Diabetic vs Control groups, *P* = 0.008; ^b^, Diabetic vs TETA-treated diabetic rats, *P* = 0.03; ^c^, Diabetic vs Control rats, *P* = 0.012.

In addition to the conventional method of obtaining individual data points for [Ca^2+^]_i_ and stress at each [Ca^2+^]_o_ value to fit by the Hill equation, we analyzed phase-plots between [Ca^2+^]_i_ and tetanic force corresponding to a series of [Ca^2+^]_o_ levels (Figure 4C). Values obtained during the rising phase of the phase-plots represented the maximum force produced by a trabecula at a particular [Ca^2+^]_i_ value. Comparisons between the slopes of the initial portion of the phase-plots from the three different experimental groups provides an indication of the dependence of cardiac contractility on [Ca^2+^]_i_. Diabetic rats had decreased slopes compared to controls (diabetic 0.7 ± 0.2, *n =* 7 vs control 2.2 ± 0.5, *n =* 9, *P* = 0.02), and TETA prevented this reduction (TETA-treated diabetic 5 ± 2.1, *n =* 7, *P* = 0.05). This finding is consistent with that obtained using the conventional Hill equation fitting method (Figure 4B, Table 2).

**Figure 5:**
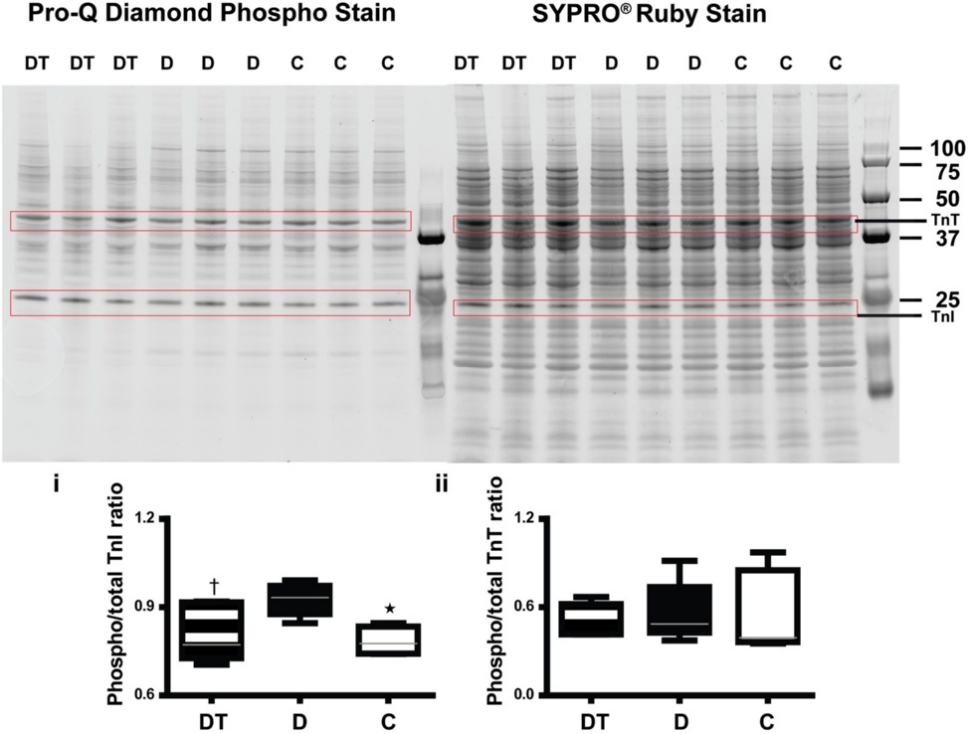
**Gel electrophoresis of LV extracts from treatment groups of rats.** Gels were stained with Pro-Q Diamond (*for phosphoproteins*) and SYPRO^®^ Ruby (*for total protein*). Upper panels show representative gels (n = 3/group). Lower panels show graphical representation of the results of densitometry: (i) Diabetic animals (D, n = 6, solid bars) showed increased proportions of phosphorylated TnI (expected molecular weight, 28 kDa) compared with controls (C, n = 6, open bars) whereas TETA-treated diabetic animals (DT, n = 6, patterned bars) showed levels comparable to control values. (ii) Neither diabetes nor TETA treatment modified the phosphorylation of TnT (expected molecular weight, 40–42.5 kDa). Data in lower panels are means ± SEM analysed by one-way ANOVA (*P* = 0.005) with *post-hoc* Tukey’s Multiple Comparisons tests: *, C vs D, *P* < 0.01; †, D vs DT, *P* < 0.05. *Abbreviations*: TnI, troponin I; TnT, troponin T.

Myofibrillar Ca^2+^ sensitivity is not only determined by the binding affinity of Ca^2+^ to troponin C (TnC) but also affected by the phosphorylation status of the troponin complex (comprising TnI, TnT, and tropomyosin), by the cooperation between the troponin complex and F-actin, and by force generation via binding of cross-bridges. Alterations in protein expression of TnT [60] and TnI [61], or changes in their phosphorylation status [62] have been reported to occur in diabetic cardiomyopathy in association with decreased myofibrillar Ca^2+^ sensitivity. Here, we found that there were no significant differences in levels of TnT or TnI between diabetic and control LV myocardium (Figure 4E). In addition, TETA had no measurable effects on the expression level of these proteins in diabetic hearts. However, the proportion of phosphorylated TnI (Figure 5i) was increased in diabetic myocardium compared with control values (diabetic vs controls, *P* < 0.01), which could contribute to their decreased myofibrillar Ca^2+^ sensitivity. By contrast, TETA treatment maintained the phosphorylation of TnI at normal levels (diabetic vs TETA-treated diabetic, *P* < 0.05), therefore preventing the reduction of the myofibrillar Ca^2+^ sensitivity. In addition, neither diabetes nor TETA treatment modified the phosphorylation of TnT (Figure 5ii).

**Figure 6:**
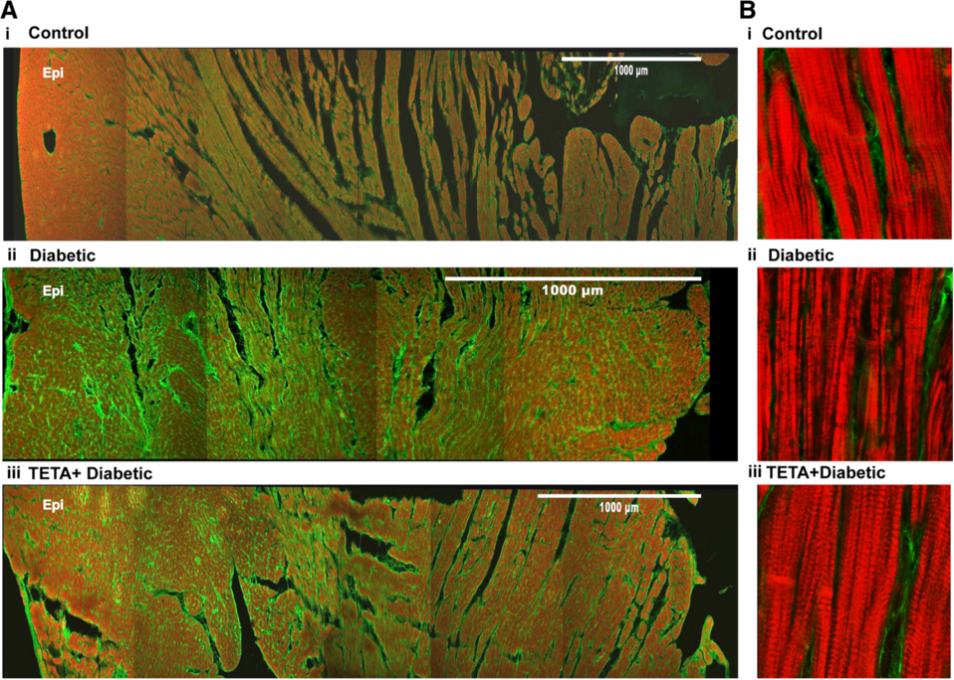
**Confocal sections of LV free wall.** Confocal microscopy was used to image fluorescent antibody labels to identify type I & type III collagen, and phalloidin-rhodamine labelled F-actin in sections (30 μm) from the LV free wall. **(A)** Shows representative LV wall cross-sections (16 х objective) labelled for F-actin (red) and type I collagen (green) in control (i), diabetic (ii) and TETA-treated diabetic rats (iii) (*n =* 7/group). Diabetic rats had decreased LV wall thickness and increased collagen labelling compared to controls; TETA-treatment normalized type I collagen labelling. **(B)** illustrates representative longitudinal sections of LV tissues (40 х objective, zoom 3) from control (i), diabetic (ii) and TETA-treated diabetic rats (iii). Diabetic rats displayed disrupted F-actin structure whereas TETA-treatment maintained F-actin integrity.

**Figure 7:**
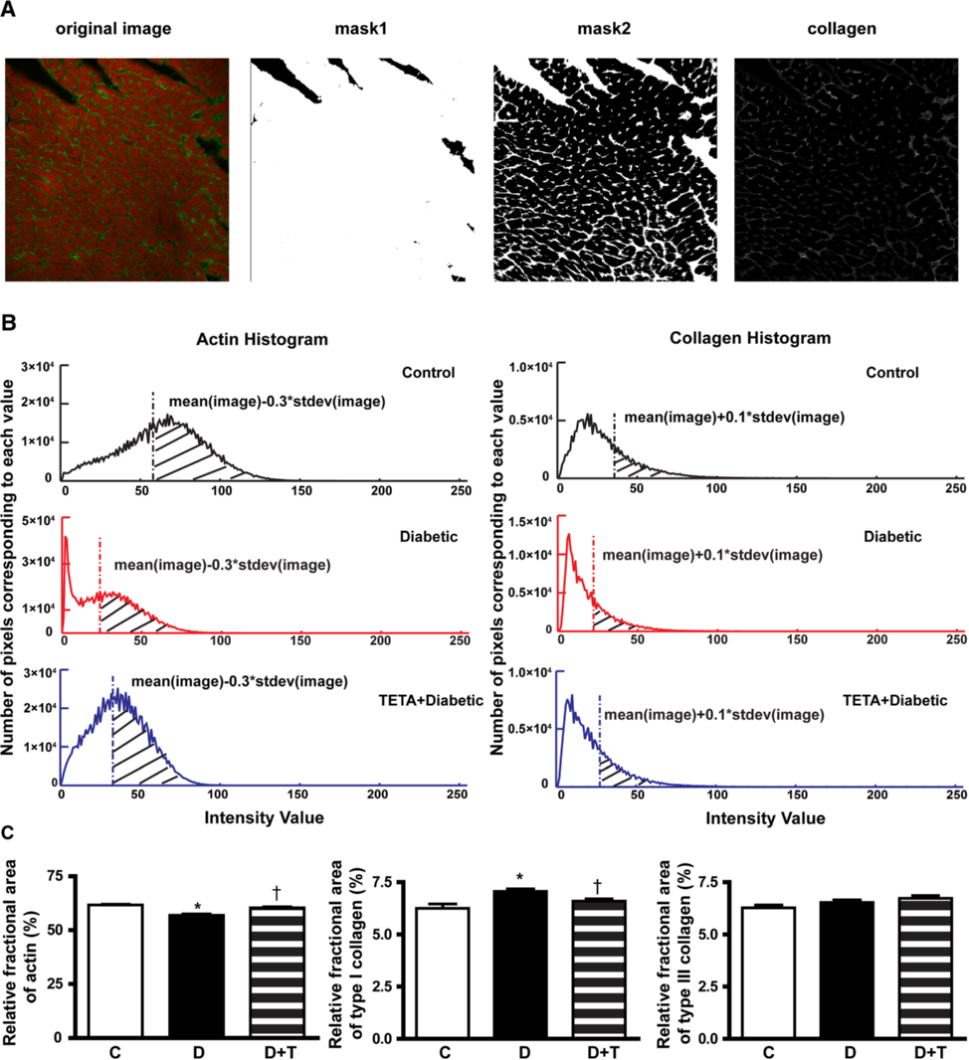
**Quantification of collagen and F-actin.** Quantification of collagen and F-actin were based on signal thresholding of matched LV endocardial areas from each group (*n =* 7 per group). To avoid possible effects of orientation and origin of the muscle fibres on the quantification, three transverse optical sections per animal were analyzed. Results have been expressed as percentages of corresponding cross-sectional areas. **(A)** A representative image illustrating application of the signal thresholding procedure. Mask1 eliminated the unlabelled area; mask2 additionally removed the F-actin labelled area. **(B)** Histogram of representative images from the three experimental groups showing the thresholds used for quantification. The criteria used for the thresholding process were maintained the same for each of the three experimental groups. The threshold setup for quantifying F-actin labelling was based on both the original image and the image following application of mask1. The threshold setup for quantifying collagen labelling was based on both the original image and the image after application of both mask1 and mask2. Shadowed areas represent signals used for quantification. **(C)** Results show that diabetic rats had increased type-I collagen content compared to controls (Diabetic: 7.1 ± 0.1% area; Control: 6.2 ± 0.2% area, *P =* 0.003), and TETA prevented this increase by retaining normal values (TETA-treated diabetic: 6.6 ± 0.1% area, *P* = 0.008). Diabetic rats also developed lowered F-actin content compared to control rats (Diabetic: 56.9 ± 0.6% area; Control: 61.7 ± 0.4% area, *P* < 0.0001); whereas, TETA-treatment prevented the lowering of F-actin values (TETA-treated diabetic: 60.4 ± 0.5% area, *P* < 0.0001). Type-III collagen did not differ significantly between groups. Data are means ± SEM, one-way ANOVA with *post-hoc* Tukey’s Multiple Comparisons test. * C vs D; † D vs D + T; *P* < 0.05.

*TETA prevented structural deterioration of cardiac muscle* Diabetic rats showed decreased LV free-wall thickness and increased intramural collagen labelling compared to control animals (Figure 6A, diabetic: 3.3 ± 0.4 mm, *n =* 8 vs control: 5.3 ± 0.3 mm, *n =* 8, *P* = 0.002). Quantification showed an average increase of 14% in type-I collagen per cross-sectional area (*P* = 0.003) in diabetic hearts, without significant change in type-III collagen area (Figure 7C). Diabetic hearts also showed disorganized F-actin (Figure 6Bii), with significantly decreased (by ~8%) content of F-actin (*P* < 0.0001, Figure 7C). TETA prevented the increase in type-I collagen labelling in diabetic hearts (*P* = 0.008, Figure 7C), and also prevented the disruption in F-actin to a significant extent (*P* < 0.0001, Figures 6Biii, 7C). These responses may account, at least in part, for TETA’s ability to prevent cardiac dysfunction.

## Discussion

In consideration of the importance of intracellular Ca^2+^ in the regulation of the cardiac contraction cycle, and reports of altered [Ca^2+^]_i_ homeostasis in diabetes [30-32], we have determined whether TETA could exert its inotropic effects through modulation of myocellular [Ca^2+^]_i_. We also sought to understand the relationship between perturbations in the myocardial homeostasis of calcium and of copper in diabetes, and the effects of TETA treatment on both.

We report here that diabetes lowered the total copper content of LV myocardium in rats by ~50% as determined by PIXE, consistent with a recent finding [63], whereas TETA-treatment restored the total myocardial copper content to normal. We also show that TETA treatment prevented the onset of cardiac dysfunction in diabetic rats despite their sustained hyperglycaemia (> 30 mmol/L), decreased body-weight and increased water consumption throughout the study, all of which are consistent with severe diabetes (Table 1).

In addition, TETA prevented the diabetes-evoked prolongation of the QT_c_ interval. QT_c_ prolongation may presage increased risk of macrovascular disease and fatal cardiac arrhythmias in diabetic patients [64], and also occurs in animals with cardiomyopathy caused by copper deficiency [13,65]. The observed correction of the QT_c_ interval in diabetic cardiomyopathy coincided with normalization of myocardial copper regulation by TETA, and could signal improvement in the electrophysiological and mechanical properties of the heart following treatment. We have previously shown that TETA treatment of non-diabetic rats, at doses equivalent to those employed in diabetic animals, had no discernible effect on cardiac structure or function [23]. Therefore, the effects of TETA treatment in non-diabetic rats were not studied further in the current work.

Here, diabetic rats showed altered [Ca^2+^]_i_ regulation as reflected in the prolonged time-to-peak [Ca^2+^]_i_ and time-constant of decay of the Ca^2+^ transient (Figure 2Bi, ii), consistent with published reports [30,31]. The measured decrease in expression of SERCA2a and its activity in these diabetic rats (Figure 3B) may well explain the slower decay of the Ca^2+^ transient. It would be expected to lower SR Ca^2+^ uptake and release, thus decreasing the amplitude of the Ca^2+^ transient. However, this effect was offset by either the prolonged action-potential duration (QT_c_ interval) observed in these diabetic rats through increasing the sarcolemmal Ca^2+^ influx; or by the increase of Ca^2+^ influx via NCX during the diastolic period due to increased [Na^+^]_i_[32]; or both. These ultimately resulted in the unchanged amplitude of the Ca^2+^ transient (Figure 2Ai, ii). Normal amplitude of the Ca^2+^ transient with slower kinetics would be expected to favour force production, but this was not the case here. On the contrary, depressed peak stress was observed in the untreated-diabetic rats. This finding is consistent with the hypothesis that altered [Ca^2+^]_i_ handling may not be the primary contributor to the depressed cardiac contractility in diabetic rats, and that TETA protects cardiac function by influencing variables other than [Ca^2+^]_i_.

Indeed, diabetic rats displayed decreased myofibrillar Ca^2+^ sensitivity (Figures 1Biii, 4D; Table 2), which may partly account for their depressed cardiac contractility, whereas TETA prevented this decrease from developing. In our study, we used intact muscle preparations to assess myofibrillar Ca^2+^ sensitivity at physiological temperature. Compared with skinned heart-muscle fibres or cells [66-69], in which the surface membrane has been permeabilized, our preparations kept the intracellular environment intact, thus avoiding the possibility that myofibrillar Ca^2+^ sensitivity was affected by perturbations in the intracellular environment. For example, disruptions of physiologically-occurring cytosolic factors such as hydrogen and phosphate ion levels would affect the sensitivity of myofilaments to Ca^2+^[68,69].

Our use of intact, ryanodine-treated LV trabeculae consistently produced tetani at different [Ca^2+^]_o_ levels (Figure 4). Upon tetanic stimulation, [Ca^2+^]_i_ rose steadily throughout whereas force plateaued and thereafter remained constant (Figure 4A), indicating that maximal Ca^2+^-activated force had been reached. At high levels of [Ca^2+^]_o_, the resting [Ca^2+^]_i_ was elevated in comparison to that at lower [Ca^2+^]_o_, suggesting the possibility of increased intracellular [H^+^]. However, when comparisons were made between the *resting* force at *high* [Ca^2+^]_o_ and the *peak* tetanic force at *low* [Ca^2+^]_o_, they were comparable for similar [Ca^2+^]_i_. For example, in Figure 4A (dashed line), the resting force at 30 mmol/L [Ca^2+^]_o_ was comparable to the tetanic force at 5 mmol/L [Ca^2+^]_o_. This indicates that high [Ca^2+^]_o_ evoked either no, or at most minor changes in intracellular [H^+^], which would be unlikely to affect trabecular force production.

Normally, tension development is initiated by Ca^2+^ binding to TnC, which triggers a series of reversible interactions and structural changes among the proteins of the thin filaments (TnI, TnT and Tm), therefore exposing the myosin binding sites on actin to enable cross-bridge formation. As a result of the cooperative nature of this process, a decreased propensity of Ca^2+^ binding to evoke the conformational changes of these proteins and cross-bridge formation would eventually affect force development. Alterations in the relative expression of TnT isoforms have been demonstrated, coincident with diminished Ca^2+^ sensitivity of skinned cardiac-muscle contractility in diabetic rats [60]. Here, we found that neither diabetes nor TETA affected cardiac levels of TnT and its isoforms (Figure 4E). This between-study difference may well have been caused by different experimental conditions and the disease stages used in different studies: for example, the animals in the above study had been diabetic for substantially longer than ours. Although unchanged expression levels of TnI have been reported in diabetic hearts with diminished Ca^2+^ sensitivity [70], consistent with this report (Figure 4E), others have reported down-regulation of TnI in conjunction with diminished Ca^2+^ sensitivity in a cardiac actomyosin system reconstituted with regulatory-protein complexes from diabetic hearts [61]. This further between-study difference may be attributable to differences in the experimental conditions. However, in our diabetic animals, the phosphorylation ratio of TnI was increased (Figure 5i) which may account for their decreased myofibrillar Ca^2+^ sensitivity. TETA treatment maintained the phosphorylation level of TnI in treated diabetic animals, therefore contributing to their normal Ca^2+^ sensitivity. The altered phosphorylation states of one or more of the contractile proteins perhaps reflect increased protein kinase C (PKC) activity [71]. This is confirmed by our previous report that myocardial PKC_δ_ gene expression is increased by ~30% in diabetic myocardium [72]. PKC_δ_ is uniquely able to phosphorylate Ser23/Ser24 in troponin I; and consequently, like protein kinase A (PKA), it can reduce myofilament Ca^2+^ sensitivity [73].

We have previously reported that, in this animal model, the expression of mRNA corresponding to β-myosin heavy-chain undergoes a ~50% increase in LV tissues, whereas that of α-myosin heavy chain is decreased to ~35% of control values [72]. However, TETA treatment had no significant effect on the expression of either α-myosin or β-myosin heavy chains in the myocardium of these diabetic animals (Cooper GJ et al., unpubl. data). This would exclude the contribution of changes in the expression of myosin isoforms to TETA’s inotropic effect.

Further, we report here that diabetes decreases total myocardial copper to markedly-lowered values, whereas we previously showed increases in the systemic and cardiac Cu(II) in the same diabetic model, as can be extracted by intra-arterial infusion of TETA. The excess Cu(II) is likely to be bound in the ECM [23,25] since copper is mainly (~95%) present in the intracellular compartment as Cu(I) and the remaining ~5% is in the extracellular space as Cu(II) [74,75]. While increased extracellular Cu(II) can promote oxidative stress by generating hydroxyl radicals [8], decreased myocardial copper can also act to cause pro-oxidant stress by impairing antioxidant defences mediated by copper-enzymes, for example lowered activity of endothelial EC-SOD and plasma ferroxidase [25,29,76]. These effects are all reversible by TETA-treatment [25,29,76]. In addition, we have shown at the protein level that TETA can lower ROS production by reversing the impaired expression of mitochondrial proteins involved in their production in diabetic LV tissue [77]. The increased oxidative stress in diabetic rats can lead to increased levels of oxidized-glutathione, further contributing to decreased Ca^2+^ sensitivity [78], which is confirmed by our previous study showing that mRNAs corresponding to several enzymes regulating aspects of glutathione metabolism were altered in diabetic cardiomyopathy [72]. Thus TETA treatment can bolster antioxidant defences in diabetic animals [29], thereby contributing to maintenance of myocardial Ca^2+^ sensitivity.

Myocardial copper deficiency is a known cause of myocardial fibrosis and cardiac failure [12,13,15,16]. Our findings suggest that the relative distribution of the two copper valence states has become dysregulated in diabetic cardiomyopathy. While total myocardial Cu level was decreased in diabetic cardiomyopathy, systemic and cardiac Cu (II) levels were increased [23,25]. This phenomenon is different from that in dietary copper restriction-induced cardiomyopathy, where both myocardial and systemic copper levels are decreased [13,15]. Interestingly, there is a report that, in a heart-specific knockout of *Ctr1*, cardiomyopathy induced by lack of myocardial copper transport led to decreased copper accumulation in cardiac tissue, accompanied by an unexpected increase in serum copper levels [79]. The authors suggested that peripheral organs may communicate with copper acquisition and storage organs through an unknown mechanism. This finding indicates the need for further studies on copper transport and its regulation in different organ systems in our diabetic animal models, to improve understanding of copper regulation in diabetes and in association with the response to TETA treatment.

Consistent with our previous reports [23,32], cardiac remodelling in diabetic rats was also observed in this study, as shown by the increased collagen content and disrupted F-actin in LV tissue (Figures 6, 7). We previously demonstrated regeneration of cardiac structure by TETA after cardiomyopathy had been present for several weeks [23]. By contrast, we show here that TETA *prevented* the development of cardiomyopathy and remodelling when administered *ab initio*. The increased collagen content and disrupted F-actin in diabetic hearts could in part account for the depressed cardiac contractility and decreased myofibrillar Ca^2+^ sensitivity observed therein (Figures 1, 2). TETA significantly prevented excessive collagen deposition and F-actin disruption, thereby maintaining muscle integrity in diabetic hearts (Figures 6, 7), which could account for its prevention of defective myocardial contractility and Ca^2+^ sensitivity. The mechanisms underlying this suppression of cardiac remodelling may be attributable to the impaired activity of endothelial EC-SOD and plasma ferroxidase in our STZ-diabetic animals. These impairments could cause myocardial fibrosis via excessive TGF-β/SMAD signaling. However, these effects are reversible by TETA-treatment [25,76]. Our previous data indicate that TETA action may suppress the pathogenic activation of a myocardial TGF-β/SMAD signalling pathway that in turn mediates the expression of genes encoding extracellular matrix (ECM) proteins and ECM-degrading enzymes [80,81], by restoration of normal EC-SOD regulation.

This study has potential limitations. Some workers have suggested that the cardiomyopathy in STZ-diabetic rats may reflect, at least in part, direct myocardial toxicity of the drug in addition to the effects of diabetes *per se*. However, we have validated the STZ-based model we used here by showing that these diabetic rats respond both to untreated diabetes and following TETA treatment, in ways that closely reflect responses in patients with T2D [21-24,28]. To date, we have shown that TETA treatment exerts substantive effects to restore LV mass in patients with LVH [24]. There are large gaps in current understanding of diabetes-mediated effects on the molecular regulation of myocardial copper homeostasis, and the molecular basis of its restoration by TETA treatment. Studies to clarify these issues are clearly required. Furthermore, there are newly described mechanisms influencing cardiomyocyte function that may act in parallel with defective copper regulation*.* For instance, Zn^2+^-mediated effects on myocardial relaxation are more pronounced in the hyperglycaemic state, possibly due an insulin-dependent effect in enhancing sequestration of [Ca^2+^]_i_ via SERCA2a [63]. The peptide obestatin, which is encoded by the ghrelin gene, up-regulates the transcription of myocardial β_1_-adrenergic receptors [82]. Moreover, alpha-lipoic acid diminishes interstitial fibrosis by decreasing collagen deposition [83]. These three effects are endogenous in mammals and may represent potentially confounding mechanisms when evaluating myocardial function. Whether any of these other factors will ultimately prove to be of clinical significance awaits the introduction of interventions based on these processes into informative clinical trials.

In this study, we have relied upon the high degree of specificity of Cu^2+^-TETA binding [25,26], as well as direct measurements of myocardial copper by PIXE, and our prior quantitative studies of systemic copper homeostasis, to support the role of copper in the processes under study. The findings we have reported here are consistent with our recent study showing TETA-mediated reversal of LV dysfunction in diabetic rats in vivo [28]. However, there are as yet no published data showing that TETA treatment can suppress overt heart failure in diabetic patients. Furthermore, in order to establish the validity of TETA treatment for this condition, it will be necessary to show that it has a clinically significant beneficial effect on important outcomes such as mortality and/or hospitalization. Such data are not yet available.

In conclusion, we report here that TETA-treatment prevented the development of diabetes-induced cardiac dysfunction without modifying elevated circulating blood glucose levels, thereby breaking the link between hyperglycaemia and myocardial damage. Our data support the hypothesis that altered [Ca^2+^]_i_ handling is unlikely to be the primary contributor to cardiac dysfunction in diabetes. The maintenance of normal cardiac contractility in TETA-treated diabetic rats may be attributable to chelator-mediated prevention of deterioration in myofibrillar Ca^2+^ sensitivity and cardiac muscle structure. The molecular basis of TETA function requires further elucidation. We would note that it is already documented to repair mitochondrial structure-function, extracellular matrix integrity and myocardial contractile machinery via restoration of myocardial copper-catalysed anti-oxidant defences.

## Abbreviations

[Ca2+]i: Intracellular ionic calcium concentration; Cu(II): Divalent copper; EC: Extracellular; ECM: Extracellular matrix; F-actin: Filamentous actin; LV: Left ventricular; Na+: Sodium; NCX: Na^+^/Ca^2+^ exchanger; Ni2+: Nickel ion; PKA: Protein kinase A; PKC: Protein kinase C; ROS: Reactive oxygen species; SERCA: Sarco/endoplasmic reticulum Ca^2+^ ATPase; SOD: Superoxide dismutase; STZ: Streptozotocin; TETA: Triethylenetetramine; TnC: Troponin C; TnI: Troponin I; TnT: Troponin T; T2D: Type-2 diabetes mellitus.

## Competing interests

The authors declare no duality of interest associated with this manuscript.

## Authors’ contributions

LZ participated in study design, acquisition and analysis of data, interpretation of results, and wrote the first draft of the manuscript; GC participated in study concept and design, data analysis and interpretation of results, and wrote manuscript drafts; AP and SZ participated in study concept and design, data analysis and interpretation of results; MW and MC participated in study design, data analysis and interpretation of results. All authors participated in critical revision and approved the final version of the manuscript. LZ and GC are the guarantors of this work and, as such, had full access to all of the data in the study and take responsibility for the integrity of the data and the accuracy of the data analysis. All authors read and approved the final manuscript.
